# Supplementary material for: Multivariate mixed-effects ordinal logistic regression models with difference-in-differences estimator of the impact of WORTH Yetu on household hunger and socioeconomic status among OVC caregivers in Tanzania
Source: PLoS One. 2024 Apr 16;19(4):e0301578. doi: 10.1371/journal.pone.0301578 (PMC11020529; doi:10.1371/journal.pone.0301578)
Supplement: S1 Table — (PDF) [file pone.0301578.s001.pdf]

S 1 Table 1. Frequency distribution of respondents at baseline and at follow-up, disaggregated by gender												
	Men				Women				All			
Characteristic	Baseline		Follow-up		Baseline		Follow-up		Baseline		Follow-up	
	n	%	n	%	n	%	n	%	n	%	n	%
**OVERALL**	74,977	100.0	74,394	100.0	174,678	100.0	173,244	100.0	249,655	100.0	247,638	100.0
**WORTH Yetu**												
Not a member	74,977	100.0	67,271	90.4	174,678	100.0	155,260	89.6	249,655	100.0	222,531	89.9
A member	0	0.0	7,123	9.6	0	0.0	17,984	10.4	0	0.0	25,107	10.1
**Gender**												
Women	—	—	—	—	174,678	100.0	173,244	100.0	174,678	70.0	173,244	70.0
Men	74,977	100.0	74,394	100.0	—	—	—	—	74,977	30.0	74,394	30.0
**Age**												
18-29 years	2,081	2.8	1,154	1.6	13,145	7.5	8,069	4.7	15,226	6.1	9,223	3.7
30-39 years	11,750	15.7	8,086	10.9	41,205	23.6	31,895	18.4	52,955	21.2	39,981	16.1
40-49 years	22,605	30.2	20,347	27.4	51,513	29.5	50,646	29.2	74,118	29.7	70,993	28.7
50-59 years	16,512	22.0	18,904	25.4	30,230	17.3	36,752	21.2	46,742	18.7	55,656	22.5
60+ years	22,029	29.4	25,903	34.8	38,585	22.1	45,882	26.5	60,614	24.3	71,785	29.0
**Marital status**												
Married or living together	54,428	72.6	54,497	73.3	71,707	41.1	79,869	46.1	126,135	50.5	134,366	54.3
Divorced or separated	8,164	10.9	8,283	11.1	29,807	17.1	29,288	16.9	37,971	15.2	37,571	15.2
widow or widower	9,569	12.8	9,323	12.5	57,605	33.0	50,825	29.3	67,174	26.9	60,148	24.3
Single or unmarried	2,816	3.8	2,291	3.1	15,559	8.9	13,262	7.7	18,375	7.4	15,553	6.3
**Education**												
Never attended	13,695	18.3	13,585	18.3	39,294	22.5	38,891	22.5	52,989	21.2	52,476	21.2
Primary	58,748	78.4	58,285	78.4	129,466	74.1	128,467	74.2	188,214	75.4	186,752	75.4
Secondary+	2,534	3.4	2,524	3.4	5,918	3.4	5,886	3.4	8,452	3.4	8,410	3.4
**HIV status**												
Negative	31,831	42.5	31,520	42.4	67,762	38.8	67,084	38.7	99,593	39.9	98,604	39.8
Positive	24,022	32.0	23,918	32.2	68,586	39.3	68,241	39.4	92,608	37.1	92,159	37.2
Unknown	19,124	25.5	18,956	25.5	38,330	21.9	37,919	21.9	57,454	23.0	56,875	23.0
**Place of residence**												
Rural	50,968	68.0	50,461	67.8	89,805	51.4	88,785	51.3	140,773	56.4	139,246	56.2
Urban	24,009	32.0	23,933	32.2	84,873	48.6	84,459	48.8	108,882	43.6	108,392	43.8

**Health insurance**												
Uninsured	65,435	87.3	62,547	84.1	154,489	88.4	145,536	84.0	219,924	88.1	208,083	84.0
Insured	9,542	12.7	11,847	15.9	20,189	11.6	27,708	16.0	29,731	11.9	39,555	16.0
**Disability status**												
Not disabled	71,816	95.8	71,244	95.8	169,741	97.2	168,341	97.2	241,557	96.8	239,585	96.8
Disabled	3,161	4.2	3,150	4.2	4,937	2.8	4,903	2.8	8,098	3.2	8,053	3.3
**Family size**												
2-3 people	47,891	63.9	47,417	63.7	108,999	62.4	107,899	62.3	156,890	62.8	155,316	62.7
4-6 people	23,079	30.8	22,982	30.9	58,881	33.7	58,594	33.8	81,960	32.8	81,576	32.9
7+ people	4,007	5.3	3,995	5.4	6,798	3.9	6,751	3.9	10,805	4.3	10,746	4.3
