# Supplementary material for: Multivariate mixed-effects ordinal logistic regression models with difference-in-differences estimator of the impact of WORTH Yetu on household hunger and socioeconomic status among OVC caregivers in Tanzania
Source: PLoS One. 2024 Apr 16;19(4):e0301578. doi: 10.1371/journal.pone.0301578 (PMC11020529; doi:10.1371/journal.pone.0301578)
Supplement: Attachment — Submitted filename: Recommendation.docx [file pone.0301578.s002.docx]

This manuscript presents an important and timely research topic that is of its kind in in many sub-regions on the African continent. Health services uptake in connection to economic needs is an important area that needs national exploration to capture relevance-embedded interventions aiming to empower community to improve livelihoods.

I have read the manuscript several times and have concluded that the authors conducted the study in a novel and appropriate manner adhering to research ethics guidelines and step-wise procedure. The methodology including the study design, settings, sampling, and data collection were thoroughly described in clear language. Only the following to take into account.

Methodology

On line 34 … the follow-up data (2019-2020) …. Was it the endline data? Can you clarify to make the reader understand better?

On line 153 it mentioned 25 Tanzania region, does the study included all regions. If not what was the exclusion criteria?

The statistical analysis in my opinion was robust and clearly presents the findings of the study that I believe many readers will understand and appreciate but I also believe that the write up of the results could be presented in a more step-by-step manners for most readers. The tables are presented very clearly nonetheless.

The discussion is well presented and reflects what the relationships of the findings are to existing literature.

Overall this is great piece of work that pave a way of evaluating embedded economic strengthening intervention to a parent intervention of improving access to social health care.
