# Supplementary material for: Multivariate mixed-effects ordinal logistic regression models with difference-in-differences estimator of the impact of WORTH Yetu on household hunger and socioeconomic status among OVC caregivers in Tanzania
Source: PLoS One. 2024 Apr 16;19(4):e0301578. doi: 10.1371/journal.pone.0301578 (PMC11020529; doi:10.1371/journal.pone.0301578)
Supplement: Attachment — Submitted filename: Response to Reviewers.docx [file pone.0301578.s003.docx]

**Response to reviewers’ comments**

**Manuscript ID**: PONE-D-23-25792 “*Multivariate mixed-effects ordinal logistic regression models with difference-in-differences estimator of the impact of WORTH Yetu on household hunger and socioeconomic status among OVC caregivers in Tanzania*”

| **Comments** | **Authors’ response** |
| --- | --- |
| **ACADEMIC EDITOR** |  |
| Your manuscript has been reviewed and the manuscript requires minor corrections before it can be submitted. Please addressing the following minor comments which have also been raised by the reviewers;  In line 34 …you mention the use of the follow-up data (2019-2020), the reader may be interested to know whether this was an endline data? Provide some bit of explanation.  Briefly explain why the mixed effects ordinal logistic regression models were the ones suited for the data.  Journal requirements:  When submitting your revision, we need you to address these additional requirements.  1. Please ensure that your manuscript meets PLOS ONE's style requirements, including those for file naming. The PLOS ONE style templates can be found at  <https://journals.plos.org/plosone/s/file?id=wjVg/PLOSOne_formatting_sample_main_body.pdf> and  <https://journals.plos.org/plosone/s/file?id=ba62/PLOSOne_formatting_sample_title_authors_affiliations.pdf>  2. Your ethics statement should only appear in the Methods section of your manuscript. If your ethics statement is written in any section besides the Methods, please move it to the Methods section and delete it from any other section. Please ensure that your ethics statement is included in your manuscript, as the ethics statement entered into the online submission form will not be published alongside your manuscript.  3. Please include a copy of Table 6 which you refer to in your text on page 22.  4. Please include captions for your Supporting Information files at the end of your manuscript, and update any in-text citations to match accordingly. Please see our Supporting Information guidelines for more information: <http://journals.plos.org/plosone/s/supporting-information>.  5. Please review your reference list to ensure that it is complete and correct. If you have cited papers that have been retracted, please include the rationale for doing so in the manuscript text, or remove these references and replace them with relevant current references. Any changes to the reference list should be mentioned in the rebuttal letter that accompanies your revised manuscript. If you need to cite a retracted article, indicate the article’s retracted status in the References list and also include a citation and full reference for the retraction notice.  Additional Editor Comments:  There are some minor comments raised by the reviewers (see them below) and once addressed the manuscript will be ready for publication. Address the comments and return the manuscript for final review | Thank you. We have addressed all the comments.  The ‘follow-up data (2019-2020)’ was not the endline. The ‘follow-up’ in this study referred to data collected at the second time from the same respondents after the baseline.  The mixed effects ordinal logistic regression models were the most appropriate for the data for the following reasons. Explanations are also included in the manuscript, lines 325–350.   - *Mixed effects*: since the data were collected as repeated measurements from the same respondents, the data lost independence due to correlation of observations from the same respondents. Therefore, a multilevel or hierarchical model that takes the correlation structure into account was the most appropriate, and this produces both fixed-effects and random-effects, hence the mixed-effects. - *Ordinal*: Both outcomes – household hunger, and SES – were ordinal, therefore necessary to recognize their structures in the models. - *Logistic regression*: this regression model is designed to handle categorical outcomes. Since categorical outcomes can be in many forms such as binary (two categories such as yes/no), multinomial (more than two unordered categories), and ordinal (ordered categories), a logistic regression model that recognizes the ordered nature of the categories of the outcome variables was appropriate in our case.   Overall, the name of the regression model (ie, mixed effects ordinal logistic regression) reflects the exact process that happened in handling the analysis.  1. The manuscript meets PLOS ONE's style requirements, including those for file naming. Thank you.  2. The ethics statement was moved to the Methods section and deleted from where it was placed before, see lines 380-391. Thank you.  3. We are sorry about this. In the manuscript, what we ha labelled as ‘Figure 6’ was supposed to read ‘Table 6’. So, this has now been corrected in the revised manuscript, see line 477. Thank you.  4. We have added captions for Supporting Information files at the end of the manuscript, see lines 880-882.  5. The reference list is complete and correct. Two more references were added while addressing the reviewers’ comments. The reviewer recommended that we add some information in the Background section about the level of Gender Inequality Index (GGI) in Tanzania. We did so, and we cited the source. Another reference was added to support the explanation of the observed differences between women and men  We have carefully addressed all the comments received and the manuscript has been revised accordingly. Changes made in the manuscript can be seen in the manuscript version with track changes. |
| **Reviewers' comments** | **Authors’ response** |
| Reviewer's Responses to Questions  **Comments to the Author** 1. Is the manuscript technically sound, and do the data support the conclusions?  The manuscript must describe a technically sound piece of scientific research with data that supports the conclusions. Experiments must have been conducted rigorously, with appropriate controls, replication, and sample sizes. The conclusions must be drawn appropriately based on the data presented.  Reviewer #1: Yes  Reviewer #2: Yes  2. Has the statistical analysis been performed appropriately and rigorously?  Reviewer #1: Yes  Reviewer #2: I Don't Know  3. Have the authors made all data underlying the findings in their manuscript fully available?  The [PLOS Data policy](http://www.plosone.org/static/policies.action#sharing) requires authors to make all data underlying the findings described in their manuscript fully available without restriction, with rare exception (please refer to the Data Availability Statement in the manuscript PDF file). The data should be provided as part of the manuscript or its supporting information, or deposited to a public repository. For example, in addition to summary statistics, the data points behind means, medians and variance measures should be available. If there are restrictions on publicly sharing data—e.g. participant privacy or use of data from a third party—those must be specified.  Reviewer #1: Yes  Reviewer #2: Yes  4. Is the manuscript presented in an intelligible fashion and written in standard English?  PLOS ONE does not copyedit accepted manuscripts, so the language in submitted articles must be clear, correct, and unambiguous. Any typographical or grammatical errors should be corrected at revision, so please note any specific errors here.  Reviewer #1: Yes  Reviewer #2: Yes  5. Review Comments to the Author  Please use the space provided to explain your answers to the questions above. You may also include additional comments for the author, including concerns about dual publication, research ethics, or publication ethics. (Please upload your review as an attachment if it exceeds 20,000 characters)  Reviewer #1: This manuscript presents an important and timely research topic that is of its kind in in many sub-regions on the African continent. Health services uptake in connection to economic needs is an important area that needs national exploration to capture relevance-embedded interventions aiming to empower community to improve livelihoods. I have read the manuscript several times and have concluded that the authors conducted the study in a novel and appropriate manner adhering to research ethics guidelines and step-wise procedure. The methodology including the study design, settings, sampling, and data collection were thoroughly described in clear language. Only the following to take into account.  Methodology On line 34 … the follow-up data (2019-2020) …. Was it the endline data? Can you clarify to make the reader understand better?  On line 153 it mentioned 25 Tanzania region, does the study included all regions. If not what was the exclusion criteria?  The statistical analysis in my opinion was robust and clearly presents the findings of the study that I believe many readers will understand and appreciate but I also believe that the write up of the results could be presented in a more step-by-step manners for most readers. The tables are presented very clearly nonetheless.  The discussion is well presented and reflects what the relationships of the findings are to existing literature.  Overall this is great piece of work that pave a way of evaluating embedded economic strengthening intervention to a parent intervention of improving access to social health care.  Reviewer #2: General: This is a very interesting study that recognizes the importance of gender equity in relation to social-economic outcomes of specific interventions. The study is timely since in Tanzania there is an increase in social economic programs targeting household members but there is no concrete plan to ensure that both men and women benefit equitably from these programs.  I have minor comments Abstract 1. It is important that the abstract clearly indicates the geographic regions where the data was collected  2. The objectives of the study need to be clearly stipulated in the abstract and toward the end of the background section of the main paper  3. It is not clear in the abstract on who the WORTH YETU group was targeting, is it women or men?  4. When the author draws a conclusion that “the design, delivery, and evaluation of such programmes should happen in a gender responsive manner, recognizing that women and men are not the same with respect to the programmes”. This statement is too general, not very clear what it means that men and women are not the same with respect to the program’. The author may consider including a more specific implication of these interesting findings.  Main paper Background 1. It would be important if the author could introduce the first paragraph by providing information on the current gender inequality index in Tanzania and some evidence on gender inequality practices as this would provide a contextual foundation of the topic. The methodological aspect could be introduced in the middle of the background section.  2. Also it is important to include the main purpose of the paper towards the end of the background section  Results The author indicate that severity of household hunger declined by 45.7% among women (aOR = 0.543 [0.524, 0.563]) and 47.5% among men (aOR = 0.525 [0.497, 0.556]). I find this to be an interesting finding. It is important to highlight any possible determinants of these differences, including gender role dynamics and contextual explanations.   Conclusion It is important for authors to indicate their advice clearly in the paper when proposing that . Overall, these findings imply that women and men are different with respect to 640 livelihood programmes, hence a need to prioritize gender as a critical dimension in the 641 design, delivery, and evaluation of the programmes”.  **Do you want your identity to be public for this peer review?** For information about this choice, including consent withdrawal, please see our [Privacy Policy](https://www.plos.org/privacy-policy).  Reviewer #1: No  Reviewer #2: No | 1. Thank you.  2. Thank you. We believe that the statistical methods applied, and the depth of statistical analysis conducted were appropriate and rigorous.  3. Thank you.  4. Thank you.  Thank you.  The ‘follow-up data (2019-2020)’ as described was not the endline. The ‘follow-up’ in this study referred to data collected at the second time from the same respondents after the baseline. The issues highlighted here was noted in line 34 which is in the abstract, but more details about the baseline and follow-up data were provided in the Methods section of the manuscript.  Yes, the study was conducted in 25 regions of Tanzania. But these are not the only regions in the country. The country has 31 regions as of 2023. Since this study was based on secondary data, we included all regions where the primary study (ie, the USAID Kizazi Kipya project) was implemented. Therefore, we have added more information regarding the exclusion criteria stating that the regions in Tanzania where the USAID Kizazi Kipya project was not implemented were excluded from this study as the necessary data were unavailable (See lines 163-164).  Thank you. Yes, the results are presented step-by-step, with informative subheading of each dimension of the findings presented.  Thank you.  Thank you.  Thank you.  Abstract  1. Thank you. We have indicated the regions in the abstract as the number of them covered by the study (line 38). Because of observing word count, we did not specifically mention them in the abstract, but they are fully described in the Methods section (lines 177-181).  2.Thank you. As instructed, we have clarified the objectives of the study in the abstract (see lines 28-33).  3.Thank you. We have clarified in the abstract that the WORTH Yetu targeted both women and men all of whom were caregivers of orphaned and vulnerable children (OVC) in Tanzania (see lines 31).  4. Thank you. For an abstract, it was not easy to provide deeper details because of word count, but so exist in the Discussion section. The main takeaway here is that gender is a critical factor in livelihood programmes and results are supportive of this. Therefore, we believe it is a strong, valid, and evidence-informed recommendation that this factor – gender – should deliberately be taken into account at all levels of such and similar programmes from as early as conception knowing that women and men are different.  Background  1. Thank you. We have added the recent (2021) Gender Inequality Index (GII) statistic for Tanzania (see lines 106-111) and cited the source. Given the focus, we maintained the methodological details in the first paragraphs of the Introduction section.  2. Thank you. The objectives are included in the Background section, see lines 127-132.  Results  Thank you so much.  We have added a few lines in the Discussion section, highlight possible determinants of the observed differences between women and men, see lines 618-623.  Conclusion  Thank you. We have revised the Conclusion section to ensure clarity, see line 667–675. |

END
